# The Wnt Signaling Pathway Inhibitors Improve the Therapeutic Activity of Glycolysis Modulators against Tongue Cancer Cells

**DOI:** 10.3390/ijms23031248

**Published:** 2022-01-23

**Authors:** Robert Kleszcz, Jarosław Paluszczak

**Affiliations:** Department of Pharmaceutical Biochemistry, Poznan University of Medical Sciences, 4, Święcickiego Str., 60-781 Poznań, Poland; paluszcz@ump.edu.pl

**Keywords:** head and neck cancer, tongue cancer, 2-deoxyglucose, lonidamine, Wnt pathway, PRI-724, IWP-O1, β-catenin, CBP, porcupine

## Abstract

Excessive glucose metabolism and disruptions in Wnt signaling are important molecular changes present in oral cancer cells. The aim of this study was to evaluate the effects of the combinatorial use of glycolysis and Wnt signaling inhibitors on viability, cytotoxicity, apoptosis induction, cell cycle distribution and the glycolytic activity of tongue carcinoma cells. CAL 27, SCC-25 and BICR 22 tongue cancer cell lines were used. Cells were treated with inhibitors of glycolysis (2-deoxyglucose and lonidamine) and of Wnt signaling (PRI-724 and IWP-O1). The effects of the compounds on cell viability and cytotoxicity were evaluated with MTS and CellTox Green tests, respectively. Apoptosis was evaluated by MitoPotential Dye staining and cell cycle distribution by staining with propidium iodide, followed by flow cytometric cell analysis. Glucose and lactate concentrations in a culture medium were evaluated luminometrically. Combinations of 2-deoxyglucose and lonidamine with Wnt pathway inhibitors were similarly effective in the impairment of oral cancer cells’ survival. However, the inhibition of the canonical Wnt pathway by PRI-724 was more beneficial, based on the glycolytic activity of the cells. The results point to the therapeutic potential of the combination of low concentrations of glycolytic modulators with Wnt pathway inhibitors in oral cancer cells.

## 1. Introduction

Altered metabolic activity is required to satisfy the increased needs of highly proliferating cancer cells. Moreover, metabolic alterations are also mechanistically related to other hallmarks of cancer. As with other tumor types, the Warburg effect, which is a typical change in energy metabolism associated with enhanced lactate production (aerobic glycolysis), was also observed in head and neck squamous cell carcinomas (HNSCC) [1]. Indeed, glucose was identified as the most important source of energy and carbon skeletons in HNSCC cells [2]. The Warburg effect can be forced via the increased activity of transcription factors, such as c-Myc and Hif-1α, which act by promoting the transcription of specific isoforms of glycolytic enzymes, e.g., hexokinase II (HKII) and pyruvate kinase M2 [1,3,4,5]. Our recent study showed the beneficial effects of c-Myc inhibition in hypopharyngeal cancer cells [6]. However, while c-Myc inhibition may block cell proliferation, it may also promote cell survival [7,8,9]; thus, it might be useful as an anticancer strategy, but rather in combination with other chemicals. Apart from c-Myc transcription factor, Akt kinase is also responsible for enhancing glucose metabolism, and the inhibition of c-Myc and Akt downregulated glucose transporters and crucial glycolytic enzymes [10]. Indeed, targeting Akt kinase seems to be a promising strategy for reducing the survival of tongue cancer cells [11].

The effective inhibition of proteins orchestrating cellular metabolism is still difficult and requires the development of appropriate small molecule inhibitors. The attenuation of excessive glucose metabolism may be reached by direct targeting of glucose flux in the glycolytic pathway. One of the crucial checkpoints of glycolysis is the activity of hexokinase catalyzing its initial step—the phosphorylation of glucose to glucose-6-phosphate. Tongue cancer cells, in comparison to noncancerous cells, are characterized by increased expression of HKII [12]. Therefore, HKII is a potentially beneficial molecular metabolic target in tongue carcinoma.

2-Deoxyglucose (2-DG) is a well-known hexokinase inhibitor [13], but its clinical use in sufficiently high doses is limited by side effects concerning the central nervous system. To bypass the problem, the 2-DG dose can be lowered, but to reach satisfactory effects, some combinatorial treatment with 2-DG should be introduced. Our previous study showed the therapeutic potential of targeting Wnt signaling in tongue cancer CAL 27 and SCC-25 cell lines [14]. Moreover, our recent study revealed a significant influence of the attenuation of Wnt signaling on glycolytic activity, which was partly potentiated by cotreatment with Akt inhibitor [11]. Indeed, the canonical Wnt pathway is often upregulated in tumor cells [15] and cancer stem cells [16]. Wnt/β-catenin signaling promotes the PI3K/Akt pathway (responsible, for instance, for the activation of energy metabolism) by tyrosine kinase receptor activation, e.g., EGFR [17]. Moreover, PI3K/Akt pathway inactivates GSK-3β kinase, leading to the increased stability of β-catenin [18]. Therefore, Wnt/β-catenin signaling and the PI3K/Akt pathway control each other in a positive feedback loop leading to the increased stability of β-catenin, which promotes the expression of Wnt pathway target genes (e.g., c-Myc), which are able to activate Hif-1α in a hypoxia-independent manner [15]. Both Hif-1α and c-Myc transcription factors upregulate the expression of glycolytic genes, which in turn induce metabolic reprogramming, known as the Warburg effect. The direct influence of Wnt signaling on the expression of glycolytic genes was observed for *MCT-1* and *LDHA* genes in colorectal cancer [19]. In addition, the inhibition of Wnt signaling by niclosamide was associated with the reduced expression of genes related to glycolysis in FaDu hypopharyngeal carcinoma cells [20].

Thus, the combination of 2-DG—a direct metabolic inhibitor—with small molecules targeting the Wnt pathway seems to be a promising strategy. Apart from 2-DG, other modulators of energy metabolism can also be useful in the context of anticancer therapy. Lonidamine is a hexokinase inhibitor that shows activity against additional molecular targets associated with energy metabolism, including the reduction of mitochondrial respiration and ATP production in cancer cells [21].

The aim of this study was to assess the effects of the combinatorial use of inhibitors of glycolysis (2-deoxyglucose and lonidamine) together with inhibitors of the Wnt pathway (PRI-724 and IWP-O1) on cell viability, proliferation and apoptosis in tongue carcinoma cells. Moreover, the effects on glycolytic activity were also assessed in order to verify whether the modulation of energy metabolism is an important contributor of the mode of action of compounds inhibiting the Wnt pathway. Based on the experimental results, we have shown that the combined inhibition of glycolysis and Wnt signaling is beneficial in the attenuation of tongue cancer cell growth.

## 2. Results

### 2.1. Wnt Signaling Inhibitors Enhance the Reduction in Cell Viability by Glycolysis Inhibitors

**Figure 1:**
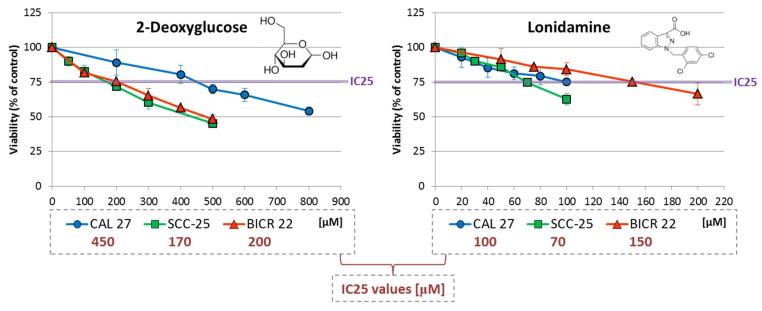
The effect of 2-deoxyglucose (2-DG) and lonidamine on the viability of tongue squamous carcinoma cell lines after 48 h of incubation, determined with an MTS assay (Promega, Madison, WI, USA). The chemical structures of the compounds are presented. The results are mean values from three independent experiments ± SD. The IC25 concentrations are shown in the legend.

First, we evaluated the effects of individual compounds (2-DG and lonidamine) on cell viability after 48 h of incubation using an MTS assay (Figure 1) and determined IC25 values, which were used in further experiments for single compounds and their combinations with Wnt signaling inhibitors.

In general, higher concentrations of 2-DG (170–450 µM) were required to reach the IC25 effect compared to lonidamine (70–150 µM). In the case of both metabolic inhibitors, SCC-25 cells were the most sensitive according to viability analysis. The highest IC25 values were shown for 2-DG in CAL 27 cells, and for lonidamine in BICR 22 cells.

IC25 values for Wnt pathway inhibitors were determined in our previous research [11]: PRI-724—2.6 µM (CAL 27), 0.85 µM (SCC-25) and 3 µM (BICR 22); and IWP-O1—1 µM (CAL 27), 10 µM (SCC-25) and 3 µM (BICR 22).

**Figure 2:**
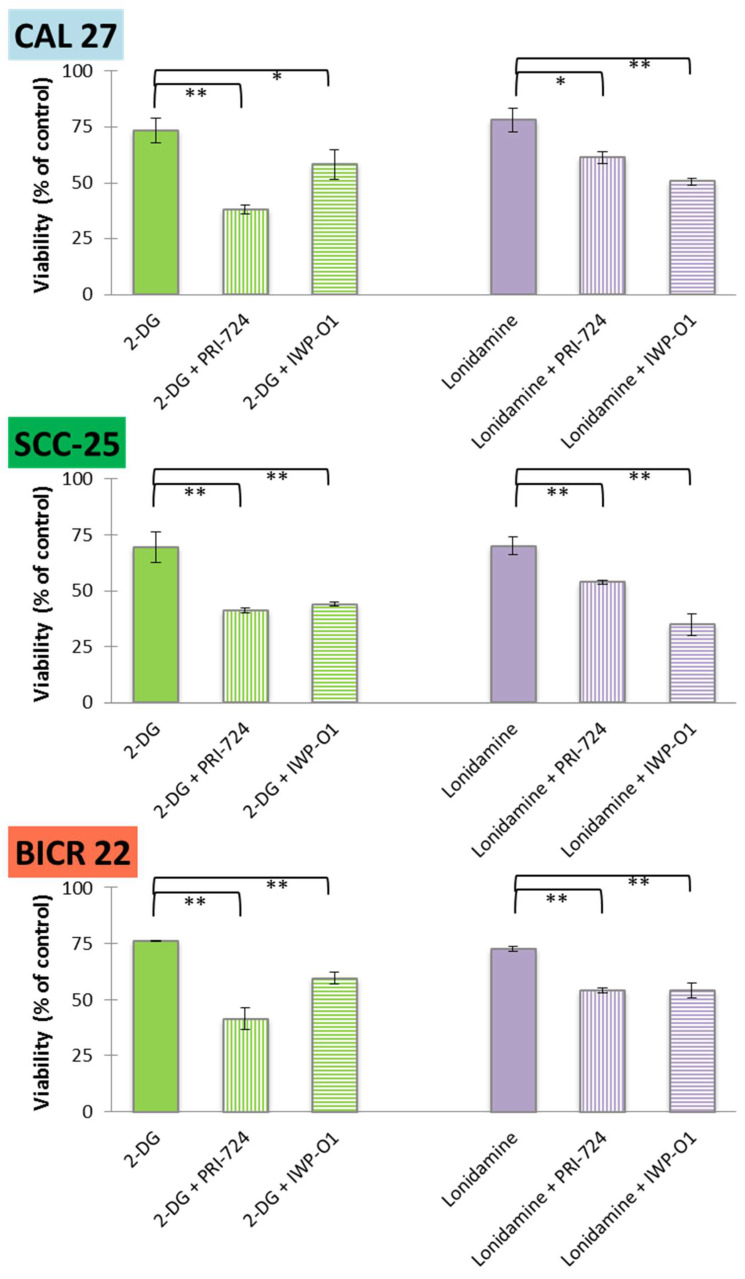
The effects of glycolytic inhibitors and their combinations with Wnt signaling inhibitors used at the IC25 concentration on the viability of tongue squamous carcinoma cell lines after 48 h of incubation, determined with an MTS assay (Promega, Madison, WI, USA). Mean values ± SD from three independent experiments are shown. * *p* < 0.05, ** *p* < 0.01, 2-DG—2-deoxyglucose.

Next, glycolytic inhibitors were applied individually (at IC25 concentrations) or in combination with Wnt inhibitors—PRI-724 or IWP-O1 (each at an IC25 concentration), and the effect of the above-mentioned combinations of chemicals (IC25 + IC25) on cell viability was evaluated using the MTS assay (Figure 2).

For all mixtures and cell lines, a significant additive reduction of viability was observed in relation to the individual effects of 2-DG or lonidamine. Moreover, viability below 50% was observed for 2-DG + PRI-724 in all cell lines, and similar results were obtained in SCC-25 cells for 2-DG + IWP-O1 and lonidamine + IWP-O1 combinations. Although CAL 27 and SCC-25 cells are both derived from primary tongue tumors, SCC-25 cells were affected by the compounds to a greater extent. For 2-DG, lonidamine and PRI-724 IC25 values for SCC-25 cells were lower comparing to CAL 27 cells. Moreover, the basal metabolic activity was higher in SCC-25 cells (see Section 2.5) than in CAL 27 cells, which may make them more sensitive to treatments modulating energy metabolism.

### 2.2. Cytotoxicity Is the Dominant Way of Action of the Compounds in BICR 22 Cells

**Figure 3:**
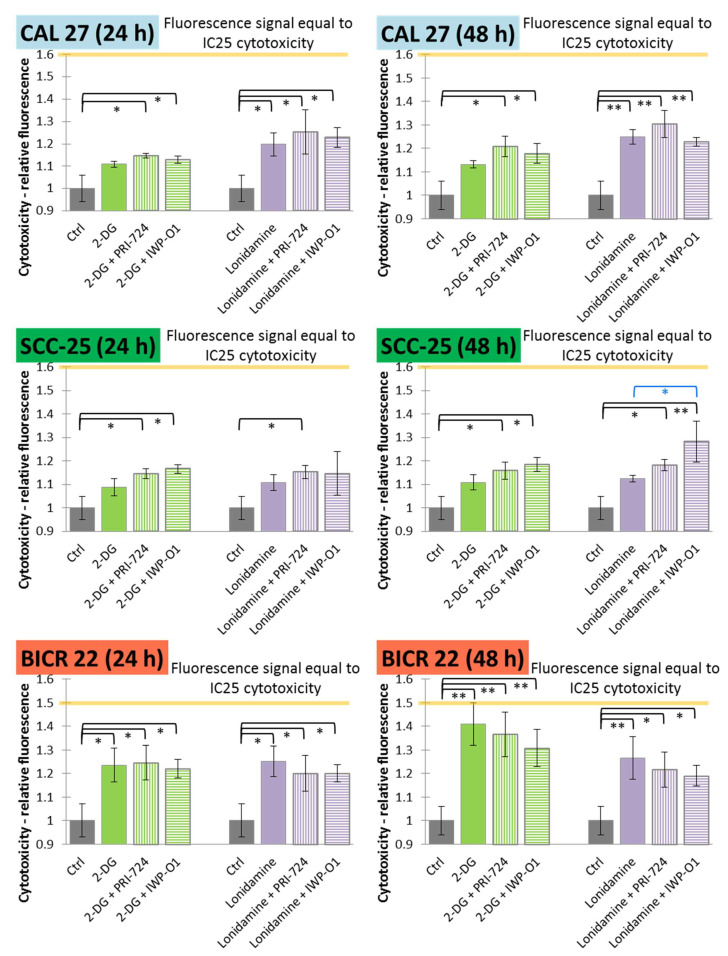
The effects of glycolytic inhibitors and their combinations with Wnt signaling inhibitors used at the IC25 concentration on the cytotoxicity of tongue squamous carcinoma cell lines after 24 and 48 h of incubation, determined with a fluorimetric CellTox™ Green Cytotoxicity Assay (Promega, Madison, WI, USA). Mean values ± SD from three independent experiments are shown. * *p* < 0.05, ** *p* < 0.01, 2-DG—2-deoxyglucose.

The MTS method, which was used for the analysis of cell viability (Figure 2), measures both the cytotoxic and antiproliferative effects of the compounds. Thus, to further assess the mechanisms of action of the chemicals used in the experiments, the analysis of cytotoxic effect (after 24 h and 48 h incubation) was introduced (Figure 3) based on the fluorimetric CellTox™ Green Cytotoxicity Assay (Promega, Madison, WI, USA).

In CAL 27 cells, significant cytotoxic effects were observed for the exclusive use of lonidamine and its mixtures with Wnt signaling inhibitors, while 2-DG was effective only in combinatorial treatment. The longer incubation time improved cytotoxic effects mostly for lonidamine and its combinations. In SCC-25 cells, neither 2-DG nor lonidamine were active in monotreatment, but in most cases the addition of Wnt pathway inhibitors enhanced the cytotoxic effects. Moreover, after 48 h incubation, the combination of lonidamine and IWP-O1 led to significantly higher cytotoxicity than lonidamine alone. In BICR 22 cells, all variants of treatment were effective and 2-DG caused the highest increase in the fluorescence signal (~41%).

Relative fluorescence values representing IC25 values of cytotoxic effects were calculated based on the positive control (relative fluorescence for cells incubated with a lysis buffer prior to measurement; see Section 4.3). The IC25 values for 24 h and 48 h of incubation are shown in Figure 3 with orange lines and are equal to 1.6 for CAL 27 and SCC-25 cells, and 1.5 for BICR 22 cells. Only for 2-DG (48 h) in BICR 22 cells was the fluorescence value close to IC25 (1.41 vs. 1.5), so in this cell line, 2-DG showed a dominant cytotoxic effect. In the other cases, fluorescence values were lower than 50% of the IC25 values.

### 2.3. IWP-O1 Potentiates Apoptosis Induction by Glycolytic Modulators in SCC-25 and BICR 22 Cells

**Figure 4:**
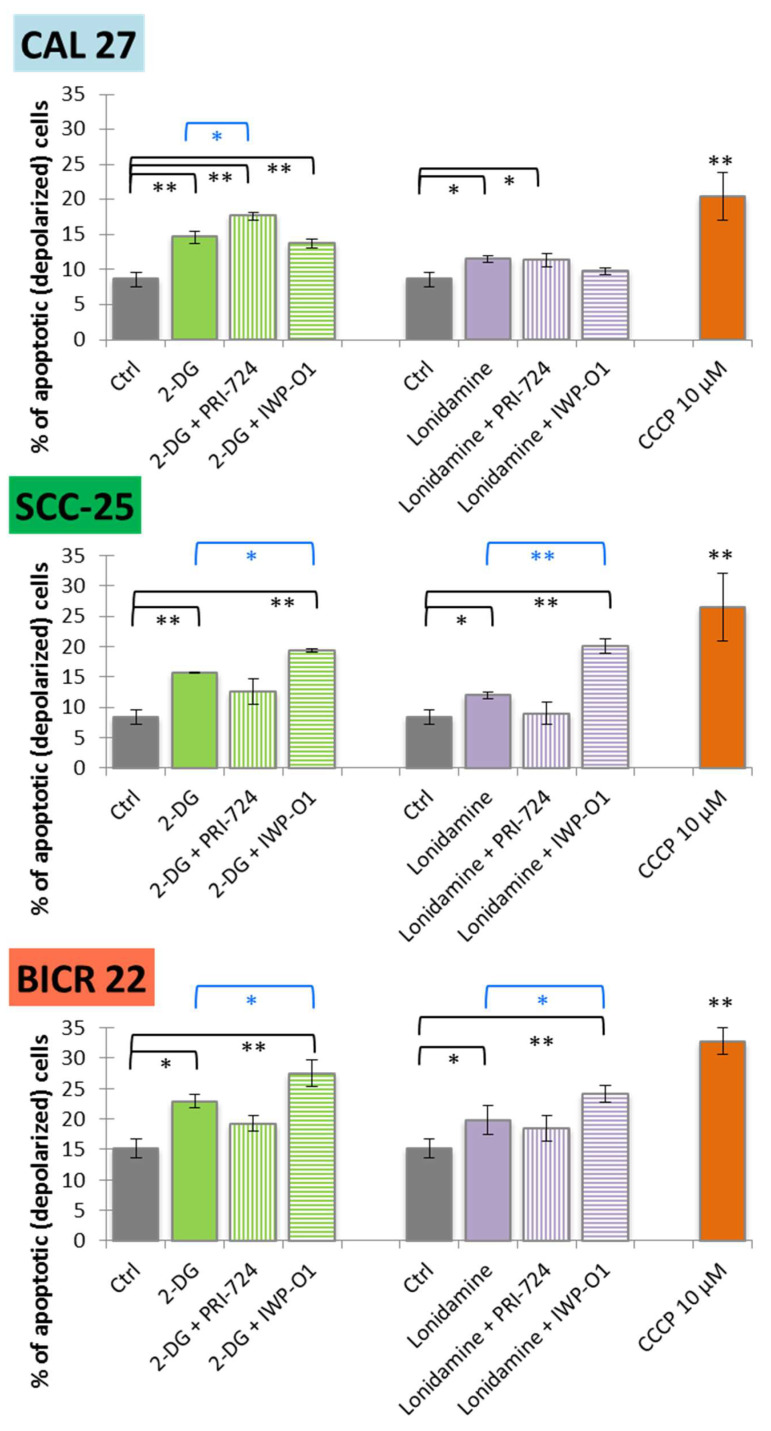
The effect of glycolytic inhibitors and their combinations with Wnt signaling inhibitors used at the IC25 concentration on apoptosis induction in tongue squamous carcinoma cell lines after 48 h of incubation. The apoptosis was measured by flow cytometric analysis of mitochondrial membrane polarization. CCCP (carbonyl cyanide 3-chlorophenylhydrazone; 10 µM) was used as a positive control. Mean values ± SD from three independent experiments are shown. * *p* < 0.05, ** *p* < 0.01, 2-DG—2-deoxyglucose.

Further, we evaluated the potential proapoptotic activity of 2-DG, lonidamine and their combinations with Wnt signaling inhibitors. Apoptosis was evaluated based on end-point analysis of the mitochondrial transmembrane potential after 48 h of incubation with the tested compounds (Figure 4).

Carbonyl cyanide 3-chlorophenylhydrazone (CCCP, 10 µM) was used as a positive control. In all cell lines, 2-DG and lonidamine slightly induced mitochondrial membrane depolarization in comparison to the untreated control cells. Significant apoptosis was also demonstrated for 2-DG and lonidamine combinations with IWP-O1 in SCC-25 and BICR 22 cells, while mixtures with PRI-724 were effective in CAL 27 cells. Among previously analyzed single Wnt signaling inhibitors, a slight increase in apoptosis was shown for PRI-724 in CAL 27 cells, and for IWP-01 in SCC-25 and BICR 22 cells [11]. Importantly, combinations with IWP-O1 in SCC-25 and BICR 22 cells, as well as 2-DG + PRI-724 in CAL 27 cells yielded significantly better results than 2-DG/lonidamine alone. Interestingly, in SCC-25 cells, the combination of 2-DG/lonidamine with PRI-724 showed a slightly lower level of apoptotic cells than 2-DG/lonidamine alone. We can speculate that pleiotropic mechanisms of action of those molecules in mixture stand behind the shift to rather antiproliferative than proapoptotic action, while in SCC-25 cells, the results mainly showed a reduction in viability related to proliferation inhibition (Figure 2).

### 2.4. The Tested Compounds May Lead to G0/G1 Cell Cycle Arrest

**Figure 5:**
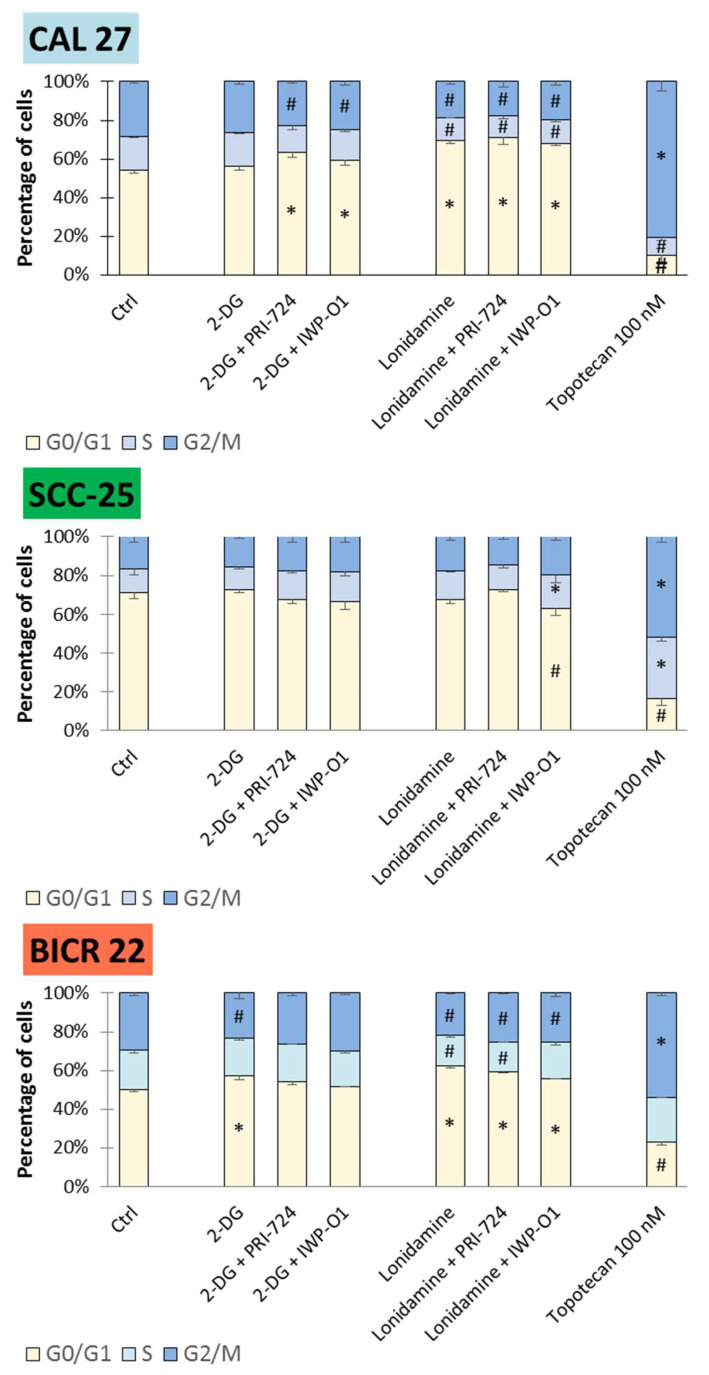
The effects of glycolytic inhibitors and their combinations with Wnt signaling inhibitors, used at the IC25 concentration on the cell cycle distribution in tongue squamous carcinoma cell lines after 48 h of incubation. The cell cycle distribution (G1/G0, S and G2/M phase) was measured by flow cytometric analysis of cells after propidium iodide staining. Topotecan (100 nM) was used as a positive control. Mean values ± SD from three independent experiments are shown. The asterisks (*) inside the bars denote a statistically significant increase in cell population between the analyzed compound used alone or in combination, and the control cells; (#) inside the bars denote a statistically significant decrease in cell population between the analyzed compound used alone or in combination, and the control cells. *p* < 0.05, 2-DG—2-deoxyglucose.

The results of the analysis of cell cycle distribution after 48 h of incubation with the compounds are shown in Figure 5.

The accumulation of cells in the G1/G0 phase with concomitant reduction in G2/M and partly S phases was observed as the dominant effect of the compounds in CAL 27 and BICR 22 cells. In detail, all combinatorial treatments and lonidamine alone affected cell cycle distribution in CAL 27 cells. Moreover, in BICR 22 cells, 2-DG, lonidamine and mixtures of lonidamine and Wnt pathway inhibitors demonstrated G1/G0 cell cycle arrest. Small changes in the cell cycle distribution for Wnt pathway inhibitors were limited to PRI-724 in CAL 27 cells and IWP-O1 in BICR 22 cells [11], and combinations of 2-DG and lonidamine show better effects. On the contrary, SCC-25 cells remained unaffected, with the exception of lonidamine + IWP-O1 treatment, where a small decrease in G1/G0 and an increase in S phases were detected.

### 2.5. PRI-724 Enhances the Metabolic Effects of Glycolytic Inhibitors

In order to confirm the modulation of energy metabolism by 2-DG or lonidamine, and also to assess the potential enhancement of such action by combining them with Wnt signaling inhibitors, we measured the effects of the chemicals on the glycolytic activity of tongue cancer cells.

**Figure 6:**
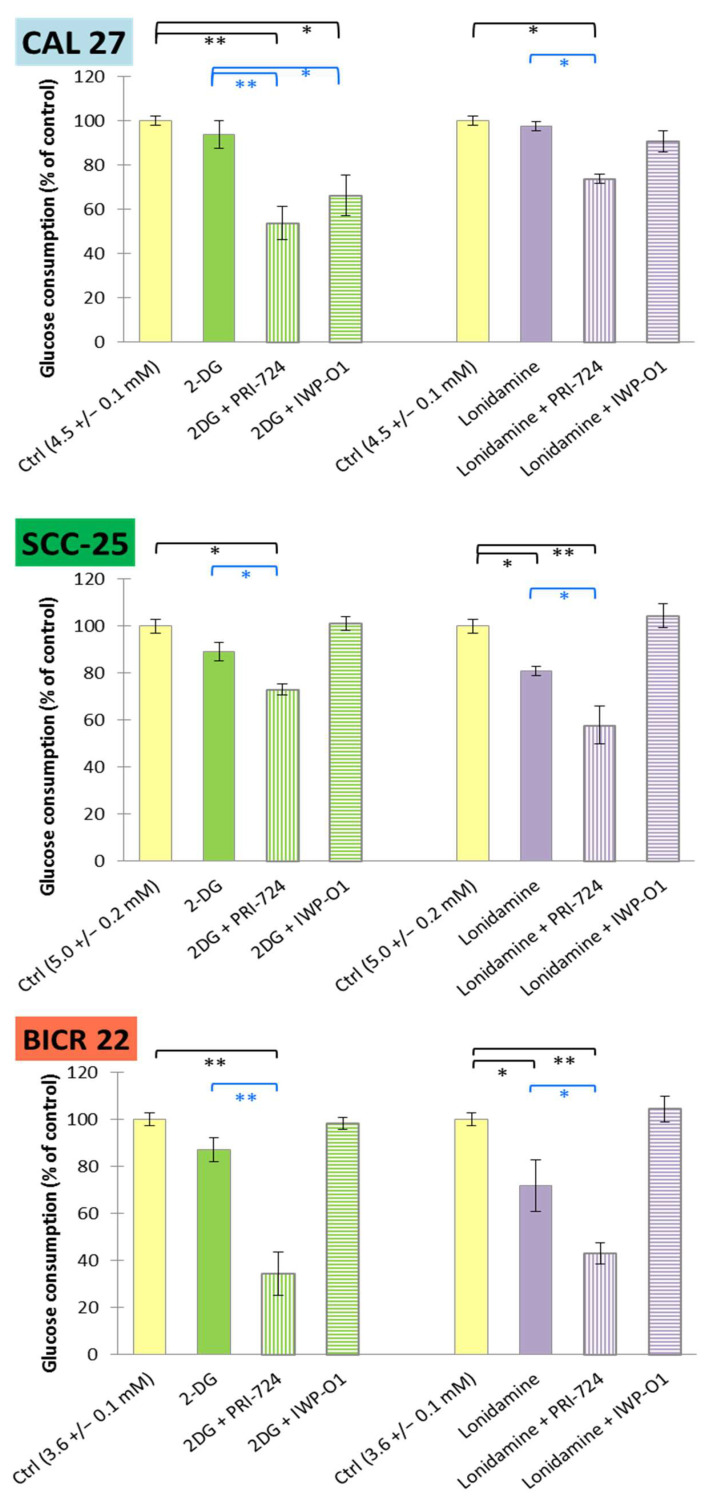
The effects of glycolytic inhibitors and their combinations with Wnt signaling inhibitors, used at the IC25 concentration on glucose consumption in tongue squamous carcinoma cell lines after 48 h of incubation. Luminescence measurements of cell culture medium were performed. Mean values ± SD from three independent experiments are shown. * *p* < 0.05, ** *p* < 0.01, 2-DG—2-deoxyglucose.

Firstly, glucose consumption was assessed via an evaluation of the reduction in glucose concentration in the culture medium after 48 h of incubation with the tested chemicals (Figure 6). The highest basal glucose consumption was detected for SCC-25 cells (5.0 ± 0.2 mM), and the lowest for BICR 22 cells (3.6 ± 0.1 mM). Lonidamine decreased its utilization by approx. 20% in SCC-25 cells and approx. 30% in BICR 22 cells, while 2-DG was insufficient for any significant reduction of glucose consumption in any of the cell lines. However, the mixtures of 2-DG or lonidamine with PRI-724 had a significant effect, reaching 60–65% reduction in BICR 22 cells.

In all cases, mixtures of 2-DG or lonidamine with PRI-724 revealed stronger reducing effects than individual metabolic modulators. The effectiveness of IWP-O1 was limited to the combination with 2-DG in CAL 27 cells, but neither IWP-O1 [11] nor 2-DG applied individually had a relevant effect on glucose consumption.

**Figure 7:**
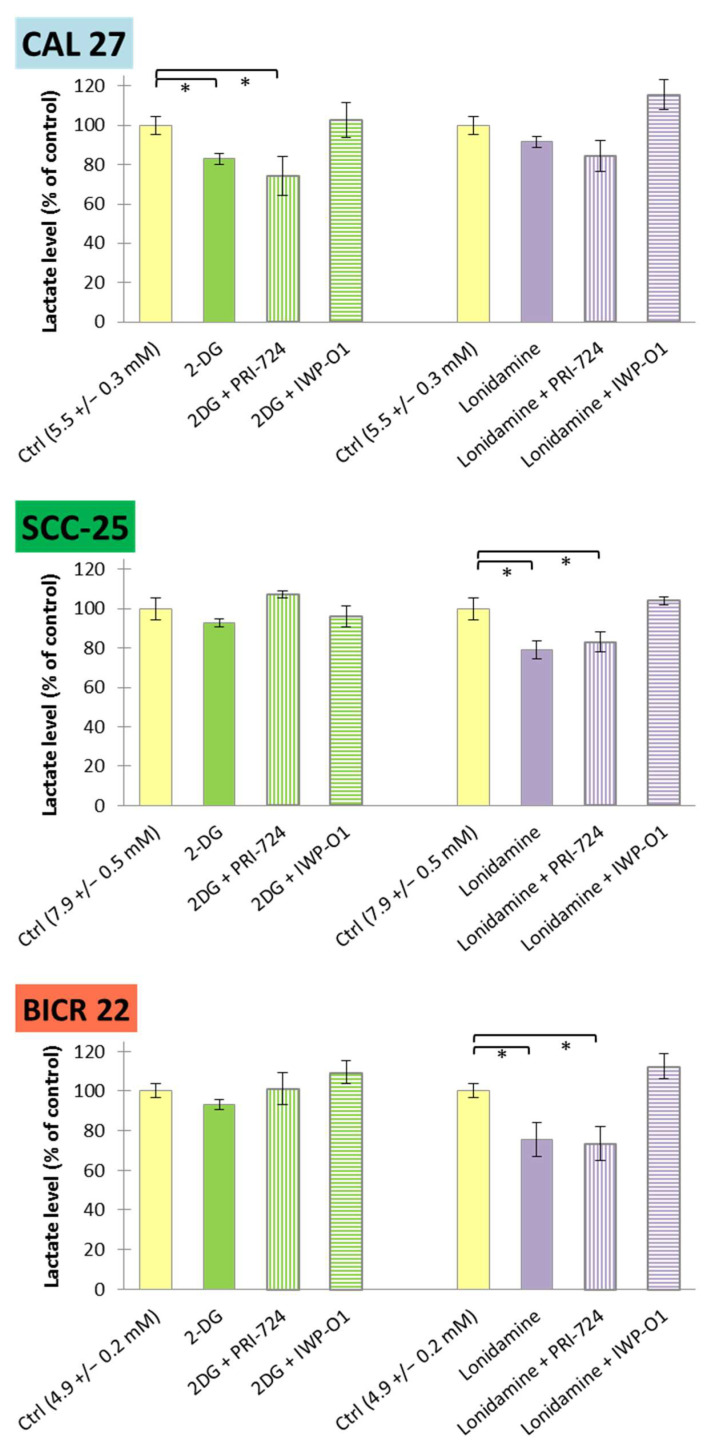
The effects of glycolytic inhibitors and their combinations with Wnt signaling inhibitors, used at the IC25 concentration on lactate release in tongue squamous carcinoma cell lines after 48 h of incubation. Luminescence measurements of cell culture medium were performed. Mean values ± SD from three independent experiments are shown. * *p* < 0.05, 2-DG—2-deoxyglucose.

Furthermore, lactate release to a culture medium was measured as a terminal marker of aerobic glycolysis (Figure 7). Similarly to glucose consumption, SCC-25 cells showed the highest basal level of lactate release (7.9 ± 0.5 mM), while BICR 22 cells showed the lowest (4.9 ± 0.2 mM). Reduced lactate release was observed for 2-DG and its combination with PRI-724 in CAL 27 cells, and for lonidamine and its mixture with PRI-724 in SCC-25 and BICR 22 cells. In our previous study, individually applied PRI-724 was found to slightly decrease lactate release in CAL 27 and BICR 22 cells, but not in SCC-25 cells [11]. Importantly, IWP-O1 combined with metabolic modulators tended to enhance lactate production.

## 3. Discussion

New effective molecular targeted therapy is needed for the successful treatment of HNSCC patients, especially those with advanced, recurrent tumors. An important role in HNSCC cells biology is played by changes in energy metabolism, including the widely known Warburg effect. In addition, the deregulation of Wnt signaling was also shown to be important for the growth of oral carcinoma [14,22], and a potential association between Wnt signaling and energy metabolism was observed [11]. In the current study, the effects of the co-inhibition of glycolysis and Wnt signaling were evaluated in tongue squamous cell carcinoma cell lines derived from primary tumors (CAL 27 and SCC-25 cells) or a lymph node metastasis of tongue cancer (BICR 22 cells). The metabolic activity of tongue cancer cells was modified by 2-deoxyglucose and lonidamine, while PRI-724 and IWP-O1 inhibitors were used for Wnt pathway modulation.

Glycolytic flux is potently decreased by 2-DG via the inhibition of hexokinase, the first and key regulatory enzyme of glycolysis. Furthermore, 2-DG reduces cellular ATP levels and antioxidant power by decreasing the flow of metabolites into the pentose phosphate pathway. Moreover, it induces autophagy and disrupts protein N-glycosylation [13]. Despite the known efficacy of 2-DG action against glycolytic flux, the main problem with the use of clinically relevant 2-DG doses is related to the depression of the central nervous system as a result of the brain’s dependence on glucose. However, it may be solved by the introduction of a ketogenic diet [23]. On the other hand, the beneficial properties of 2-DG could be achieved by using lower doses in combination with other chemicals to improve the therapeutic effects. The addition of 2-DG to standard chemo- and radiotherapy can enhance sensitivity of cells in vitro, but more potent antimetabolites are required for in vivo use [24]. Cotreatment of FaDu cells with 2-DG and cisplatin enhanced cytotoxic effects with concomitant increased oxidative stress [25], while in Jurkat cells, the combination with borasertib or everolimus induced apoptosis without an elevation in reactive oxygen species production [26]. In contrast, 2-DG seems to decrease the efficacy of anti-EGFR therapy by erlotinib application in a xenograft model of HNSCC tumors, possibly via induction of cytoprotective autophagy [27]. In our study, the comparison of the results of the viability and cytotoxicity tests revealed that the effects of 2-DG in BICR 22 cells are predominantly related to cytotoxic response induction, while in the two other tumor cell lines the effects were related mostly to reduced proliferation. This is in agreement with a slight accumulation of cells in the G1/G0 phase. In all three cell lines, 2-DG increased the number of apoptotic cells by 6–8% compared to DMSO treated cells after 48 h of incubation, so even the IC25 concentrations seemed to be able to induce the programmed death of tongue cancer cells.

Lonidamine is an example of another hexokinase inhibitor, for which several mechanisms of action were proposed. Similarly to 2-DG, it attenuates the flux of metabolites through glycolysis and the pentose phosphate pathway, but additionally it inhibits mitochondrial respiration by blocking succinate–ubiquinone reductase activity of respiratory complex II and mitochondrial pyruvate carrier [21]. Moreover, lonidamine causes intracellular tumor acidification as a result of inhibition of MCT-1, 2 and 4 lactate transporters [21,28]. The limitation of the clinical use of lonidamine is predominantly related to its toxicity against the liver, which may be reduced by using liposome formulations of this compound [29]. Antiglycolytic therapy based on lonidamine in combination with other chemicals may be more advantageous [29,30]. The reduction in cell viability by lonidamine in CAL 27 and BICR 22 cells resulted from the induction of cytotoxic and antiproliferative effects. In comparison to other chemicals used in monotherapy, lonidamine caused the most effective accumulation of cells in the G1/G0 phase in BICR 22 cell lines, but apoptosis induction was weaker than for 2-DG.

The modulation of canonical Wnt signaling was shown to alter energetic metabolism, e.g., in colorectal cancer cells [19,31]. In a previous study we observed that the inhibition of the Wnt canonical pathway can be efficient in lowering glycolytic activity in oral cancer cells, including SCC-25 cells, in which it enhanced the effect of the Akt inhibitor [11]. In this study, we evaluated the potential benefits of combinatorial treatment with metabolic modulators and Wnt signaling inhibitors. PRI-724 targets the nuclear part of the Wnt/β-catenin-dependent pathway via disruption of the interaction between β-catenin and CBP histone acetyltransferase [32], while IWP-O1 targets porcupine—the *O*-acyltransferase crucial for maturation of Wnt ligands—thus attenuating both β-catenin-dependent and independent pathways [33]. We previously described the benefits of Wnt pathway inhibition in HNSCC [6,20,34,35]; however, the simultaneous targeting of Wnt signaling and other molecular pathways is necessary for the potentiation of the anticancer effects.

In this study, we performed experiments using combinations of glycolytic inhibitors—2-DG or lonidamine—with small molecule inhibitors of the Wnt signaling pathway—PRI-724 or IWP-O1—at low (IC25) concentrations. In general, the results showed significant improvement of the observed effects after the application of mixtures in comparison to single 2-DG/lonidamine use. Beneficial results were observed for viability, cytotoxicity, apoptosis and cell cycle analysis, and only some differences were reported for mixes of 2-DG and lonidamine with PRI-724/IWP-O1. Comparing particular cell lines, SCC-25 cell viability was more effectively inhibited by combinations of metabolic and Wnt signaling inhibitors than CAL 27 cells. Moreover, IC25 concentrations of 2-DG, lonidamine and PRI-724 were lower in SCC-25 cells. In previous research assessing the migration potential of CAL 27 and SCC-25 cells [14], the migration of unaffected SCC-25 cells was higher, similar to its higher basal glycolytic activity in the current research. Thus, SCC-25 cells appear to show higher proliferation, metabolic rate and motility. This can partly be the reason for SCC-25 cells’ higher susceptibility to viability reduction by the compounds. Moreover, the pleiotropic mechanism of action of 2-DG/lonidamine mixtures with PRI-724 may be responsible for the appearance of an antiproliferative, rather than proapoptotic, effect in this cell line. On the other hand, CAL 27 cells were also affected, e.g., in regard with apoptosis induction and cell cycle inhibition, so beneficial effects were shown for both primary tongue cancer cell lines. Moreover, in metastatic BICR 22 cells, we observed the highest cytotoxic effect, which shows the potential of this strategy in the search for a beneficial treatment of more advanced tumors.

Metabolic analysis revealed important differences in the actions of Wnt inhibitors. PRI-724 in combination with 2-DG/lonidamine significantly reduced glucose consumption by tongue cancer cells, and decreased lactate release to a culture medium. In turn, IWP-O1—an inhibitor of porcupine—had no significant effect on glucose consumption, and tended to increase lactate production, with the exception of its mixture with 2-DG in CAL 27 cells. That suggests a differential influence on energy metabolism, depending on whether only the canonical or both the canonical and noncanonical Wnt signaling are affected. The molecular mechanisms of these phenomena need to be further elucidated.

## 4. Materials and Methods

### 4.1. Cell lines and Culture

Three commercially available tongue squamous cell carcinoma cell lines were used in this study: CAL 27 and SCC-25 cell lines (derived from primary tumors) were purchased from American Type Culture Collection (ATCC), while BICR 22 cells (derived from a lymph node metastasis) were purchased from European Collection of Authenticated Cell Cultures (ECACC).

The CAL 27 and BICR 22 cells were grown in a high-glucose DMEM medium (Biowest, Nuaillé, France), supplemented with 10% FBS (EURx, Gdańsk, Poland) and 1% antibiotic solution (penicillin and streptomycin; Biowest, Nuaillé, France). SCC-25 cells were grown in a 1:1 mixture of DMEM medium with F12 medium containing 1.2 g/L sodium bicarbonate, 2.5 mM L-glutamine, 15 mM HEPES and 0.5 mM sodium pyruvate (Biowest, Nuaillé, France) supplemented with 10% FBS (EURx, Gdańsk, Poland), 1% antibiotics solution (penicillin and streptomycin; Biowest, Nuaillé, France) and 400 ng/mL hydrocortisone (Sigma-Aldrich, St. Louis, MI, USA). Cells were cultured under standard conditions (37 °C and 5% CO_2_) in a humidified incubator (Memmert, Schwabach, Germany).

### 4.2. Chemicals and MTS Viability Assay

Four small-molecule inhibitors were used in the experiments. 2-Deoxyglucose (2-DG) and lonidamine (Sigma-Aldrich, St. Louis, MI, USA) were used to directly modulate energy metabolism. Metabolic modulators were combined with Wnt signaling inhibitors—PRI-724 (Selleck Chemicals, Pittsburgh, PA, USA) and IWP-O1 (Sigma-Aldrich, St. Louis, MI, USA). Each time directly before use, 2-DG was dissolved in PBS buffer, and stock solutions of the other compounds were prepared in DMSO and stored in aliquots at −20 °C.

The MTS assay was performed in order to assess the effect of single compounds on the viability of CAL 27, SCC-25 and BICR 22 cells, and to determine IC25 values. Then, viability reduction after treatment with combinations of chemicals at equipotent concentrations (IC25 + IC25) was evaluated. The assay was performed using CellTiter 96^®^ AQueous One Solution Reagent (Promega, Madison, WI, USA) according to the manufacturer’s protocol. Briefly, the cells were seeded in 96-well plates (10^4^ cells per well) and left for 24 h preincubation. Then, the appropriate growth medium was replaced with fresh medium containing various concentrations of the tested compounds and the cells were incubated for an additional 48 h. The cells incubated with solvent (DMSO not exceeding 0.2% or PBS) served as a control (100% viability). Afterwards, the wells were rinsed with PBS buffer and fresh medium containing MTS solution (100 µL + 20 µL) was added. After 60 min of incubation, the absorbance at 490 nm was read using an Infinite M200 multiplate reader (Tecan, Grödig, Austria). The assay was performed in triplicate, each time with four replicates per assay. The IC25 values, which were determined based on the MTS assay, were used in further experiments for single glycolysis inhibitors and their combinations with Wnt signaling inhibitors (IC25 + IC25).

### 4.3. Cytotoxicity Assay

The CellTox™ Green Cytotoxicity Assay (Promega, Madison, WI, USA) was used to assess the cytotoxic effects of single compounds and their combinations. The assay, which measures the changes in membrane integrity by detecting the binding of a fluorescent dye to DNA, was performed according to the manufacturer’s recommendations. Briefly, the cells were seeded in black 96-well plates (10^4^ cells per well) and left for 24 h preincubation. Then, the appropriate growth medium was replaced with fresh medium (100 µL per well) containing IC25 concentrations of glycolysis inhibitors or their combinations with Wnt signaling inhibitors and the cells were incubated for an additional 24 or 48 h. The cells incubated with solvent served as a negative control. Additionally, the cells that were incubated 30 min before end of the experiment with lysis solution (4 µL per well) served as a positive control of cytotoxic effects. Then, freshly prepared dye solution in assay buffer was directly added (100 µL per well), plates were orbitally shaken for 1 min, incubated for 15 min at room temperature in the dark, once again orbitally shaken for 1 min and the signal (ex/em −500/530 nm) was measured in an Infinite M200 multiplate reader (Tecan, Grödig, Austria). The assay was performed in triplicate, each time with three replicates per assay.

Relative fluorescence values representing IC25 values of cytotoxic effects (as the fold of control, which is equal to 1) were calculated based on the fluorescence of the positive control using the equation:

(1) $$\mathrm{IC}25\;\left(\mathrm{fold}\;\mathrm{of}\;\mathrm{control}\right)=\frac{\left(\mathrm{positive}\;\mathrm{control}\;\mathrm{RFU}\;-\;\mathrm{negative}\;\mathrm{control}\;\mathrm{RFU}\right)\cdot 25\%}{\mathrm{negative}\;\mathrm{control}\;\mathrm{RFU}}+1$$

where RFU denotes Relative Fluorescence Units.

### 4.4. Mitochondrial Transmembrane Potential

Proapoptotic effects of single compounds and their combinations were analyzed by the measurement of mitochondrial membrane potential (ΔΨm) using the Muse^®^ MitoPotential Kit (Merck, Darmstadt, Germany) according to the manufacturer’s recommendations. Briefly, 2 × 10^5^ cells per well were seeded in 6-well plates and preincubated for 24 h. Then, the appropriate growth medium was replaced with fresh medium (2 mL per well) containing IC25 concentrations of the tested glycolysis inhibitors or their combinations with Wnt signaling inhibitors, and the cells were incubated for an additional 48 h. The cells incubated with solvent served as a negative control. Additionally, cells incubated with 10 µM CCCP (carbonyl cyanide 3-chlorophenylhydrazone; Sigma-Aldrich, St. Louis, MI, USA) served as a positive control of mitochondrial membrane depolarization. Afterwards, the cells were collected by trypsinization, resuspended in 100 µL of assay buffer, 100 µL of MitoPotential working solution and incubated for 20 min at 37 °C in a humidified CO_2_ incubator. Fluorescence was analyzed by flow cytometry on a Muse^®^ Cell Analyzer (Merck, Darmstadt, Germany). Data were evaluated using Muse^®^ 1.5 analysis software (Merck, Darmstadt, Germany). All the experiments were done in triplicate.

### 4.5. Cell Cycle Analysis

The effect of single compounds and their combinations on cell cycle distribution was analyzed using the Muse^®^ Cell Cycle Kit (Merck, Darmstadt, Germany) based on propidium iodide interaction with DNA. Briefly, 2 × 10^5^ cells per well were seeded in 6-well plates and preincubated for 24 h. Then, the appropriate growth medium was replaced with fresh medium (2 mL per well) containing IC25 concentrations of the glycolysis inhibitors or their combinations with Wnt signaling inhibitors, and the cells were incubated for an additional 48 h. The cells incubated with solvent served as a negative control. Additionally, cells incubated with 100 nM topotecan (Sigma-Aldrich, St. Louis, MI, USA) served as a positive control of cell cycle arrest. Then, cells were collected by trypsinization, washed with PBS buffer and fixed in ice-cold 70% ethanol. After at least overnight storage at −20° C, fixed cells were collected by centrifugation and washed with PBS buffer. The distribution of cells, depending on the cell cycle phase (G1/G0, S, G2/M), was analyzed with the Muse^®^ Cell Analyzer (Merck, Darmstadt, Germany) after 30 min incubation with propidium iodide solution in the presence of RNase A at room temperature in the dark. Data were analyzed using Muse^®^ 1.5 analysis software (Merck, Darmstadt, Germany). All the experiments were done in triplicate.

### 4.6. Glucose Concentration Analysis

The analysis of the glucose concentration in the culture medium was performed using the Glucose-Glo™ Assay (Promega, Madison, WI, USA). Briefly, the cells were seeded in 96-well plates (10^4^ cells per well) and left for 24-h preincubation. Then, the appropriate growth medium was replaced with fresh medium containing IC25 concentrations of the glycolysis inhibitors or their combinations with Wnt signaling inhibitors, and the cells were incubated for an additional 48 h. Cells incubated with solvent served as a negative control. Afterwards, 2 µL of medium from each well was diluted in 1998 µL of PBS buffer. Luminometric analysis of the glucose concentration was performed on a GloMax^®^ Discover microplate reader (Promega, Madison, WI, USA) after 60 min incubation of diluted culture medium (50 µL per well) with a glucose detection reagent (50 µL per well). The glucose concentration in the media was determined based on a linear standard curve, which was prepared in parallel (Supplementary Figure S1). Glucose consumption was calculated as the difference between glucose concentration in cell-free media and media samples obtained after 48 h of cell incubation in the presence of the compounds. Data were normalized based on the viability assay results. The assay was performed in triplicate, each time with three replicates per assay.

### 4.7. Lactate Concentration Analysis

The analysis of the lactate concentration in the culture medium was performed using the Lactate-Glo™ Assay (Promega, Madison, WI, USA). Briefly, the cells were seeded in 96-well plates (10^4^ cells per well) and left for 24-h pre-incubation. Then, the appropriate growth medium was replaced with fresh medium containing IC25 concentrations of the glycolysis inhibitors or their combinations with Wnt signaling inhibitors and the cells were incubated for an additional 48 h. Cells incubated with solvent served as a negative control. Afterwards, 10 µL of medium from each well was diluted in 990 µL of PBS buffer. Luminometric analysis of the lactate concentration was performed on a GloMax^®^ Discover microplate reader (Promega, Madison, WI, USA) after 60 min incubation of the diluted culture medium (50 µL per well) with lactate detection reagent (50 µL per well). The lactate concentration in the media was determined based on a linear standard curve, which was prepared in parallel (Supplementary Figure S2). Lactate release was calculated as the level of lactate present in media samples obtained after 48 h of incubation. Data were normalized based on the viability assay results. The assay was performed in triplicate, each time with three replicates per assay.

### 4.8. Statistical Analysis

For the analysis of the significance of differences between controls, glycolysis inhibitors and their combinations with Wnt signaling inhibitors, the one-way ANOVA test with the Tukey post hoc test were performed, with *p* < 0.05 considered as significant. The analyses were performed using STATISTICA 11 software.

## 5. Conclusions

The use of combinations of glycolytic flux inhibitors, 2-DG and lonidamine, with Wnt signaling small-molecule inhibitors leads to beneficial anticancer effects in tongue squamous cell carcinoma. In fact, 2-DG and lonidamine might not be considered sufficiently effective in treating oral cancer cells at concentrations that are safe for the patient, and proper dosing for combinatorial treatments must be further developed in in vivo studies. Interestingly, although the activity of PRI-724 and IWP-O1 concerning cell viability, apoptosis and cell cycle distribution is similar, their influences on glucose metabolism differ. Further studies are necessary to describe the exact mechanisms of Wnt pathway-dependent modulation of glycolysis, but a reduction in glucose utilization and a decrease in lactate release suggest the benefits of PRI-724 application.

## Data Availability

Data are contained within the article and Supplementary Materials.

## Supplementary Materials

The following are available online at https://www.mdpi.com/article/10.3390/ijms23031248/s1.
