# Supplementary material for: The Wnt Signaling Pathway Inhibitors Improve the Therapeutic Activity of Glycolysis Modulators against Tongue Cancer Cells
Source: Int J Mol Sci. 2022 Jan 23;23(3):1248. doi: 10.3390/ijms23031248 (PMC8835497; doi:10.3390/ijms23031248)
Supplement: Supplementary file 1 [file ijms-23-01248-s001.zip › ijms-1543272-supplementary.pdf]

Supplementary materials

The Wnt signaling pathway inhibitors improve the therapeutic activity of glycolysis modulators against tongue cancer cells

Robert Kleszcz [1,*] and Jarosław Paluszczak [1]

1 Department of Pharmaceutical Biochemistry, Poznan University of Medical Sciences, 4, Święcickiego Str., 60-781 Poznań, Poland; kleszcz@ump.edu.pl (R.K.), paluszcz@ump.edu.pl (J.P.)

* Correspondence: kleszcz@ump.edu.pl; Tel.: +48 618546624

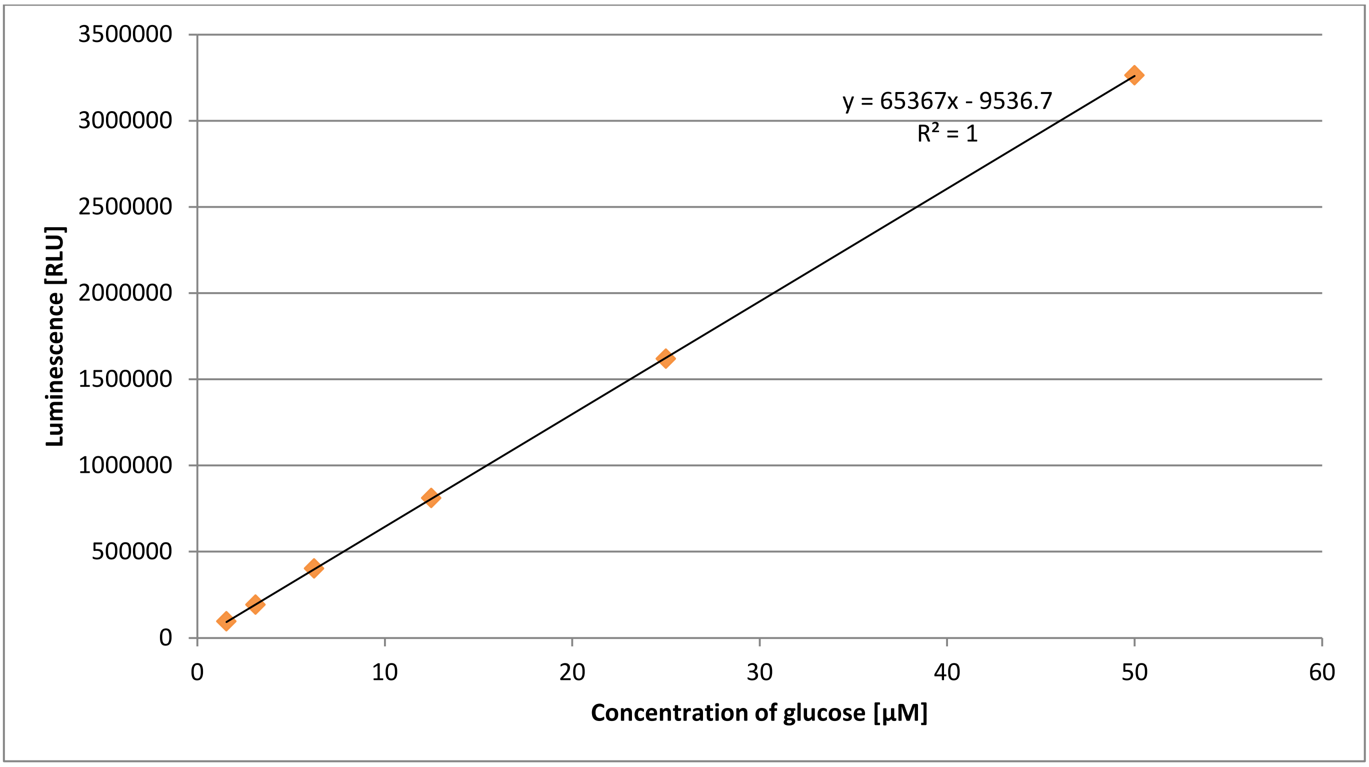

Figure S1. Standard curve for glucose concentration analysis in Glucose-Glo™ Assay.

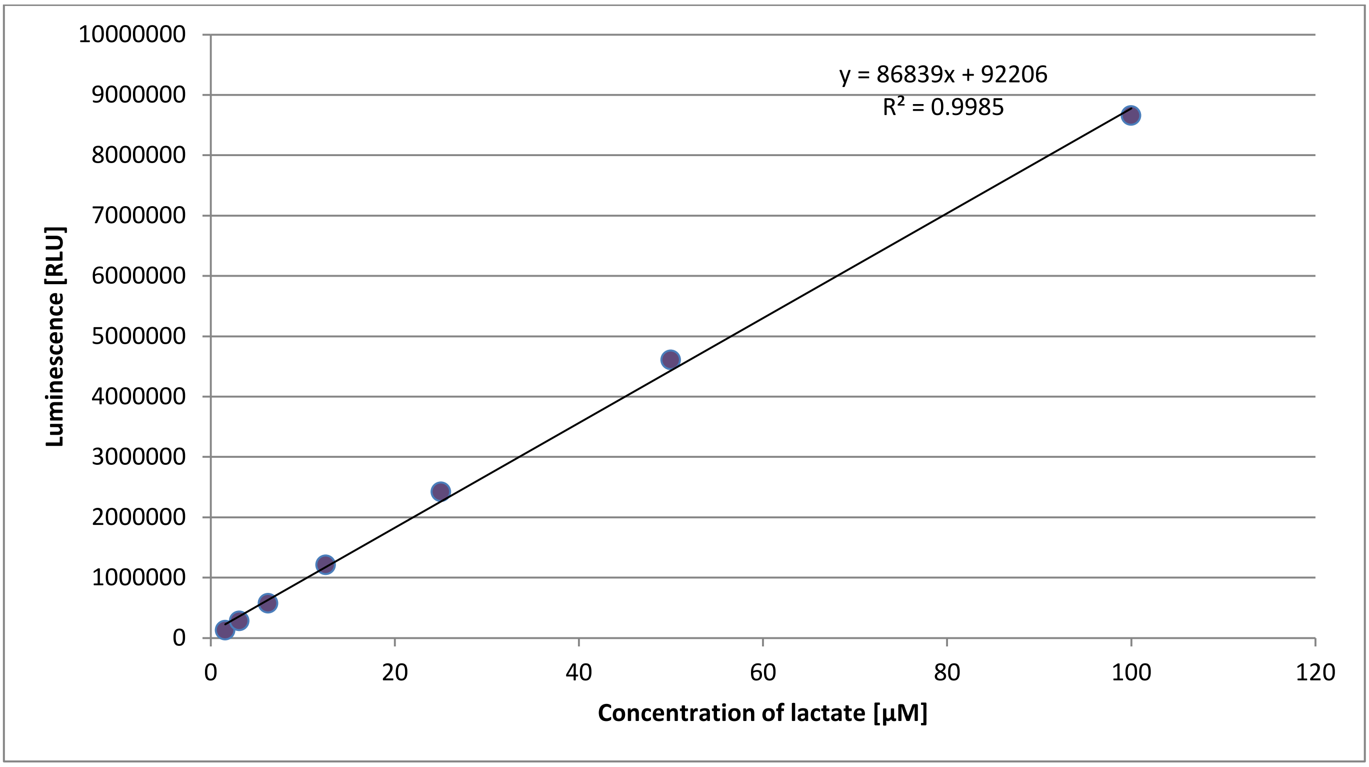

Figure S2. Standard curve for lactate concentration analysis in Lactate-Glo™ Assay.
